# Response to: Local social vulnerability as a predictor for cancer-related mortality among US counties

**DOI:** 10.1093/oncolo/oyae327

**Published:** 2025-03-26

**Authors:** Krista Y Chen, Amanda L Blackford, Ramy Sedhom, Arjun Gupta, S M Qasim Hussaini

**Affiliations:** School of Medicine, Johns Hopkins University, Baltimore, MD 21205, United States; Division of Hematology and Oncology, Perelman School of Medicine, University of Pennsylvania, Philadelphia, PA 19104, United States; Division of Hematology and Oncology, Perelman School of Medicine, University of Pennsylvania, Philadelphia, PA 19104, United States; Penn Center for Cancer Care Innovation, Abramson Cancer Center, University of Pennsylvania, Philadelphia, PA 19104, United States; Masonic Cancer Center, University of Minnesota, Minneapolis, MN 55455,United States; Department of Medicine, Division of Hematology and Oncology, O’Neal Comprehensive Cancer Center, University of Alabama at Birmingham, Birmingham, AL 35233,United States

We thank Mr. Uduba and his co-authors for their thoughtful response to our report examining associations between county-level social vulnerability index (SVI) and age-adjusted cancer mortality rates (AAMRs) across the USA.^1^

One concern was the selection of SVI data for analysis, given that there are multiple SVI datasets (2014, 2016, and 2018) available within our study period of 2013-2019. The study period was chosen based on publicly available data from the Center for Disease Control Wide-ranging Online Data for Epidemiologic Research Database at the time of publication. We subsequently selected the SVI index from 2018 as it aggregates data from the American Community Survey across the 5-year period between 2014 and 2018.^2^ Each update accordingly aggregates data from the 5 years prior, such that the 2016 update includes data from 2012 to 2016 and the 2014 update includes data from 2010 to 2014.^3,4^ Although none directly correspond to our study period of 2013-2019, the 2018 update includes only years considered in our study and centers around the same midpoint of 2016. Based on the suggestion presented by Uduba et al. to use the mid-period data, the 2018 SVI update is best suited for our analysis.

**Figure 1.**
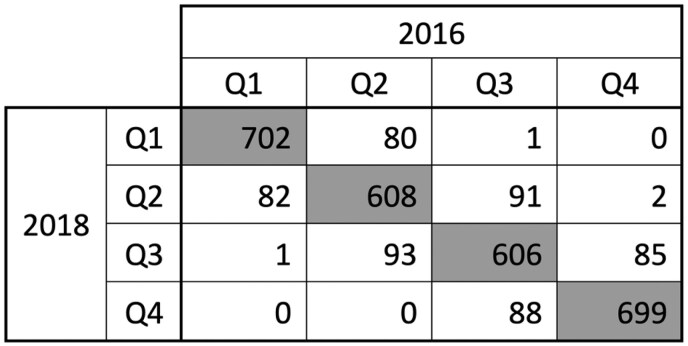
Social vulnerability index (SVI) across 2016 and 2018 updates. We classified county SVI estimates into quartiles based on their distribution among 3138 US counties for each update. Quartile 1 includes counties with SVI index 0.00-0.2499, Quartile 2 includes 0.25-0.4999, Quartile 3 includes 0.50-0.7499, and Quartile 4 includes 0.75-1.00. Five of the 3143 total US counties were excluded because data were available in only 1 SVI update or because county lines changed throughout the data collection period.

To further evaluate our methodology, we also compared SVI data from 2016 and 2018. SVI is presented as a percentile, with all US counties ranked from 0 to 1 and 1 being most vulnerable. We generated a modified SVI for both years by aggregating percentile ranks for each of the SVI components except for the Minority Status and Language component, and we present the number of counties that were classified into each quartile in Figure 1. Overall, there was 83% agreement in SVI quartile. Of the 17% of counties that moved across quartiles between the 2 updates, >99% moved only 1 quartile, corresponding to a median difference in SVI between years of −0.0005. These data suggest that the results of our study are likely robust to changes based on SVI year.

An additional concern was the exclusion of minority status and the creation of a modified SVI for analysis. Our analysis compares the highest and lowest SVI quartiles of US counties across multiple sociodemographic characteristics, including ethnicity and race. SVI is comprised of multiple variables grouped into 4 themes, including Minority Status and Language in the 2018 update. We followed the methodology of Khan et al. in removing the minority theme in order to prevent it from influencing mortality estimates when comparing across race and ethnicity.^5^ We would like to clarify that we removed the entire minority theme, including both minority status and decreased English proficiency, from the analysis. We then created the modified, composite SVI by summing the relative contributions of variables from the 3 other themes and re-generating percentile rankings for overall social vulnerability. We apologize for any imprecise phrasing used in the methodology but believe that by excluding this theme, we have limited some of the potential bias in results for minority populations.

Lastly, we acknowledge the typographical error in Figure 1 and apologize for any confusion this may have caused. We have contacted the editorial team to address this mistake and ensure that Figure 1A, showing a map of SVI quartiles, and Figure 1B, showing a map of AAMR quartiles, correspond to the correct labels within the legend.

In summary, we thank Uduba et al. for highlighting these points for clarification. We believe our methodology to be consistent with their concerns and well-designed to support our conclusions—that counties with greater social vulnerability have higher cancer mortality and that these disparities are more pronounced among individuals aged 45-65, among Hispanic populations, in southern regions, and in rural counties.
